# Alkhurma Hemorrhagic Fever Virus in *Ornithodoros savignyi* Ticks

**DOI:** 10.3201/eid1301.061094

**Published:** 2007-01

**Authors:** Rémi N. Charrel, Shamsudeen Fagbo, Gregory Moureau, Mohammad Hussain Alqahtani, Sarah Temmam, Xavier de Lamballerie

**Affiliations:** * Université de la Méditerranée, Marseille, France; † King Abdul Aziz University, Jeddah, Saudi Arabia

**Keywords:** Biosafety level 4, Saudi Arabia, flavivirus, tick, arthropodborne, arbovirus, dispatch

## Abstract

Evidence for the tickborne nature of Alkhurma hemorrhagic fever virus (AHFV) is indirect because AHFV has not been detected in arthropods. One *Ornithodoros savignyi* tick from Saudi Arabia contained AHFV RNA. This is the first direct evidence that AHFV is a tickborne flavivirus and confirms the association between human AHFV cases and tickbite history.

Alkhurma hemorrhagic fever virus (AHFV) is a recently described virus within the genus *Flavivirus*. AHFV was discovered in 1995 in a patient with hemorrhagic manifestations and fever in Saudi Arabia (*1*). Subsequently, ≈20 symptomatic patients infected with this virus have been documented by virus isolation in this country. The clinical picture is extremely severe and the case-fatality rate is >30%, which makes AHFV one of the most deadly flaviviruses (*2*). Previous studies have determined that AHFV is a variant genotype of Kyasanur Forest disease virus, another biosafety level (BSL) 4 virus that causes viral hemorrhagic fever in certain regions of India (*3*). Accordingly, AHFV is classified as a BSL-3 or BSL-4 agent, depending on country regulations.

To date, AHFV has been isolated only from human samples. Genetic and serologic characterization has grouped AHFV with tickborne flaviviruses (*1*,*3*). A previous study associated AHFV transmission to humans with butchering of sheep and camels (*1*). However, no direct evidence for its association with ticks, such as viral detection in or isolation from ticks, has been documented. To investigate the tickborne nature of AHFV, ticks were collected in western Saudi Arabia and tested by reverse transcription–PCR (RT-PCR) for AHFV.

## The Study

A total of 124 ticks were collected from camels and camel resting places in 3 different locations in western Saudi Arabia. The epidemiologic characteristics of the 124 ticks are shown in Table 1. Ticks were stored in individual containers at room temperature in Saudi Arabia and killed by overnight freezing at −80°C the day before shipment to France, according to the French regulations for importation. Samples were treated as previously described (*4*), and 200 μL of clarified, crushed material was used for purification of total nucleic acid with the MagNA Pure LC system (Roche Diagnostics, Meylan, France). Pools of 10 RNA samples (5 μL each) were prepared and tested by 1-step RT-PCR assay with the Access RT-PCR system (Promega. Madison, WI, USA) by using primers ALK-ES1 and ALK-ER (*2*). We used a cycling profile of 48°C for 45 min, 95°C for 5 min, followed by 40 cycles of 94°C for 15 s, 55°C for 30 s, and 68°C for 30 sec, and a final elongation step at 68°C for 7 min. The 10 specimens in the RT-PCR–positive pool were tested individually for confirmation and sample identification. PCR products were then sequenced.

**Table 1:** Epidemiology of ticks collected in Saudi Arabia

Location	Type of location	Ticks isolated	No. ticks	Date of collection
Northeast Jeddah	Camels and camel resting place	*Ornithodoros savignyi* nymphs and adults and *Hyalomma* spp.	64	Jun 2004
Southeast Jeddah	Camels and camel resting place	*O. savignyi* nymphs and adults and *Hyalomma* spp.	32	Jun 2004
Kilaakh (50 km from Taif)	Camels	*H. dromedarii*	28	Jan 2005

The tick JE7, which was collected southeast of Jeddah, Saudi Arabia, was positive for AHFV, and sequence analysis showed 99.7% homology at the nucleotide level with AHFV strain 1176 (GenBank accession no. AF331718) in the homologous region of the envelope gene. For safety reasons, virus isolation was not attempted in our BSL-3 laboratory. To identify the tick species, morphologic studies and molecular identification were conducted. All 124 ticks were identified by using morphologic keys (*5*).

The complete coding sequence of AHFV-JE7 was determined by amplification and sequencing of overlapping PCR products by using the long PCR product sequencing strategy (*6*) (primers and detailed protocol available on request). The open reading frame sequence was 10,248 nt (GenBank accession no. DQ154114). Pairwise distance comparison with AHFV prototype strain 1176 sequence showed 0.80% and 0.70% divergence at the nucleotide and amino acid level, respectively. A total of 79 nt substitutions were observed, of which 54 mutations were synonymous and 25 mutations were nonsynonymous (Ka:Ks ratio 0.46) (Table 2). The AHFV-JE7 complete sequence has been used with other full-length amino acid sequences of other mammalian tickborne flaviviruses to reconstruct phylogenetic relationships.

**Table 2:** Genetic differences between Alkhurma hemorrhagic fever virus coding sequences of human (strain 1176 AF331718) and tick (strain JE7, DQ154114) origin*

Gene	No. mutated sites/no. sites in gene (%)		Ka:Ks
	Synonymous mutations	Nonsynonymous mutations	
VirC	2/291 (0.69)	–	–
CTHD	–	1/60 (1.67)	–
prM	2/267 (0.75)	–	–
M	4/225 (1.78)	1/225 (0.45)	0.20
E	6/1,488 (0.41)	2/1488 (0.13)	0.25
NS1	4/1,062 (0.32)	3/1,062 (0.28)	0.43
NS2a	8/687 (1.16)	1/687 (0.15)	0.11
NS2b	2/393 (0.51)	3/393 (0.76)	0.60
NS3	9/1,863 (0.48)	5/1,863 (0.27)	0.36
NS4a	2/378 (0.53)	2/378 (0.53)	0.50
2K	1/69 (1.45)	–	–
NS4b	4/756 (0.53)	4/756 (0.53)	0.50
NS5	10/2,709 (0.37)	3/2,709 (0.15)	0.11

*Ka:Ks, ratio of nonsynonymous-to-synonymous nucleotides; VirC; mature virion C protein; CTHD, C-terminal hydrophobic domain; prM, premembrane; M, membrane; E, envelope; NS, nonstructural; 2K, transmembrane domain.

No nonsynonymous mutations were observed in the envelope gene of the 11 sequences determined for human AHFV cases or in tick and human AHFV sequences. Ka:Ks ratios within human sequences (n = 11) and between tick and human sequences were 0.33 and 0.50 in the nonstructural protein 3 (NS3) gene and 0.46 and 0.33 in the NS5 gene, respectively. However, these values were not significantly different when sequences were analyzed by χ^2^ test. Because of the low genetic heterogeneity observed between AHFV sequences, envelope, NS3, and NS5 sequences of the 12 AHFV isolates were colinearized and aligned with homologous sequences of AHFV and related tickborne flaviviruses to infer phylogenetic relationships, as previously reported (Figure ) (*2*).

**Figure:**
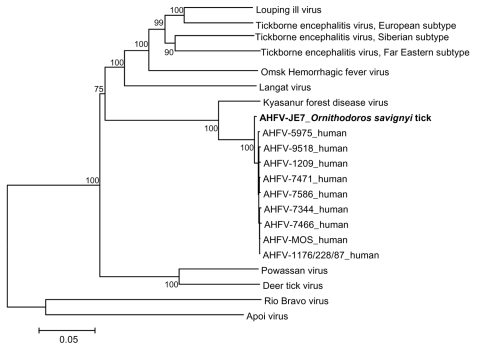
Phylogenetic analysis of AHFV-JE7 (shown in **boldface**) detected in an *Ornithodoros savignyi* tick and homologous sequences of related mammalian tickborne flaviviruses based on colinearized nucleotide sequences. Distances and groupings were determined by the p-distance algorithm and neighbor-joining method. Bootstrap values are indicated and correspond to 500 replications. Rio Bravo and Apoi viruses were used to root the tree. The scale bar at the lower left indicates a genetic distance of 0.05-nt substitutions per position.

As shown in the Figure, the AHFV-JE7 sequence was closely related to but unambiguously distinct from all other AHFV sequences from human isolates. AHFV-JE7 constituted a phylogenetic group distinct from human isolates and was supported by a 100% bootstrap value. The genetic distance of AHFV-JE7 from the common ancestor of AHFV isolates is less than that of any of the human isolates. This topology strongly suggests that human isolates are derived from the group including AHFV-JE7. This constitutes a strong argument for the tickborne nature of AHFV.

It could be argued that several mosquitoborne flaviviruses have been isolated from ticks, e.g., Saint Louis encephalitis virus and West Nile virus (*7**,**8*). Phylogenetic analysis showed that these viruses are closely related to other *Culex*-associated viruses and that their evolution is determined by mosquitoes, not by ticks. Thus, the evolution of AHFV is clearly determined by ticks because all the most closely related viruses are also known to be associated with ticks, although in at least 1 case (Powassan virus) there is some evidence indicates that the virus is also found in mosquitoes (*9*). Because ticks blood-feed on vertebrates, detection of AHFV RNA could be due to AHFV in the blood of the camel; however, this virus may not replicate in the tick. Because no obvious sign of blood was noted in the JE7 tick, detection of a full-length open reading frame sequence is convincing evidence that the virus had replicated in the tick, thus increasing the likelihood that the tick is a vector for the virus.

## Conclusions

In the arid ecosystems of Saudi Arabia and other parts of the Persian Gulf, *Ornithodoros savignyi*, the sand tampan, has been associated with camels, their resting places, and to a lesser extent, other domestic and wild animals (*5*) found in camel resting places. *O*. *savignyi* is a multiple-host–seeking, nocturnally active, cryptic tick that commonly attacks humans and other animals resting under trees (*10*), which supports its role as a vector and transmitter of AHFV. The closely related Kyasanur Forest disease virus, which is endemic in certain regions of India, is the only tickborne hemorrhagic fever virus that has been isolated from *Ornithodoros* spp. (*11*). AHFV is the first human pathogenic RNA virus to be detected in *O*. *savignyi*. Recent reports of mosquito transmission of AHFV (*12*) remain unverified (*13*) but merit investigation by virologic analyses of field-collected mosquito pools. Our study provides the first unequivocal evidence that AHFV is a tickborne flavivirus, confirms previous phylogenetic analysis linking human AHFV isolates to a tick source, and shows the value of molecular techniques in rapidly and safely detecting arboviral activity in local arthropod fauna.

Wide-ranging implications arise from the identification of *Ornithodoros* ticks, a well-established genus in the Persian Gulf region in the transmission cycle of AHFV. First, the ability of the unfed tick to remain dormant in harsh conditions for long periods may give it an extended reservoir role for AHFV. Second, the wide distribution of *Ornithodoros* ticks in the region suggests that the geographic limits of AHFV may be larger than presently assumed. A recent clinical case from Najran, which is >600 km from the Jeddah–Makkah area, supports this view (*14*). This finding reinforces the need for physicians in the region and elsewhere to consider AHFV in the etiology for undifferentiated fever.

The relationship between *Ornithodoros* ticks and military activity–related tickborne disease incidence has been recently documented in the Middle East (*15*). A large number of foreign military and civilian personnel are presently based in the region, thus expanding the opportunity for exporting clinical disease or infected ticks. The association of JE7 with camels further supports the role of camels in AHFV transmission cycle as well as the zoonotic nature of the disease. Larger studies involving more tick species are needed to better understand AHFV ecology and transmission dynamics. Investigations to obtain better knowledge of the geographic distribution of AHFV are necessary in countries near Saudi Arabia.
